# Robustness and reproducibility for AI learning in biomedical sciences: RENOIR

**DOI:** 10.1038/s41598-024-51381-4

**Published:** 2024-01-22

**Authors:** Alessandro Barberis, Hugo J. W. L. Aerts, Francesca M. Buffa

**Affiliations:** 1 Nuffield Department of Surgical Sciences, Medical Sciences Division, University of Oxford, Old Road Campus Research Building, Roosevelt Drive, Oxford, OX3 7DQ UK; 2 Computational Biology and Integrative Genomics Lab, Department of Oncology, Medical Sciences Division, University of Oxford, Oxford, OX3 7DQ UK; 3 Artificial Intelligence in Medicine (AIM) Program, Mass General Brigham, Harvard Medical School, Boston, MA USA; 4 Radiation Oncology and Radiology, Dana-Farber Cancer Institute, Brigham and Women’s Hospital, Harvard Medical School, Boston, MA USA; 5 Radiology and Nuclear Medicine, GROW & CARIM, Maastricht University, Maastricht, The Netherlands; 6 Cardiovascular Imaging Research Center, Massachusetts General Hospital, Harvard Medical School, Boston, MA USA; 7 AI and Systems Biology, IFOM ETS, 20139 Milan, Italy; 8 Department of Computing Sciences and Bocconi Institute for Data Science and Analytics (BIDSA), Bocconi University, 20100 Milan, Italy

**Keywords:** Machine learning, Software, Statistical methods

## Abstract

Artificial intelligence (AI) techniques are increasingly applied across various domains, favoured by the growing acquisition and public availability of large, complex datasets. Despite this trend, AI publications often suffer from lack of reproducibility and poor generalisation of findings, undermining scientific value and contributing to global research waste. To address these issues and focusing on the learning aspect of the AI field, we present RENOIR (REpeated random sampliNg fOr machIne leaRning), a modular open-source platform for robust and reproducible machine learning (ML) analysis. RENOIR adopts standardised pipelines for model training and testing, introducing elements of novelty, such as the dependence of the performance of the algorithm on the sample size. Additionally, RENOIR offers automated generation of transparent and usable reports, aiming to enhance the quality and reproducibility of AI studies. To demonstrate the versatility of our tool, we applied it to benchmark datasets from health, computer science, and STEM (Science, Technology, Engineering, and Mathematics) domains. Furthermore, we showcase RENOIR’s successful application in recently published studies, where it identified classifiers for SET2D and TP53 mutation status in cancer. Finally, we present a use case where RENOIR was employed to address a significant pharmacological challenge—predicting drug efficacy. RENOIR is freely available at https://github.com/alebarberis/renoir.

## Introduction

**Figure 1:**
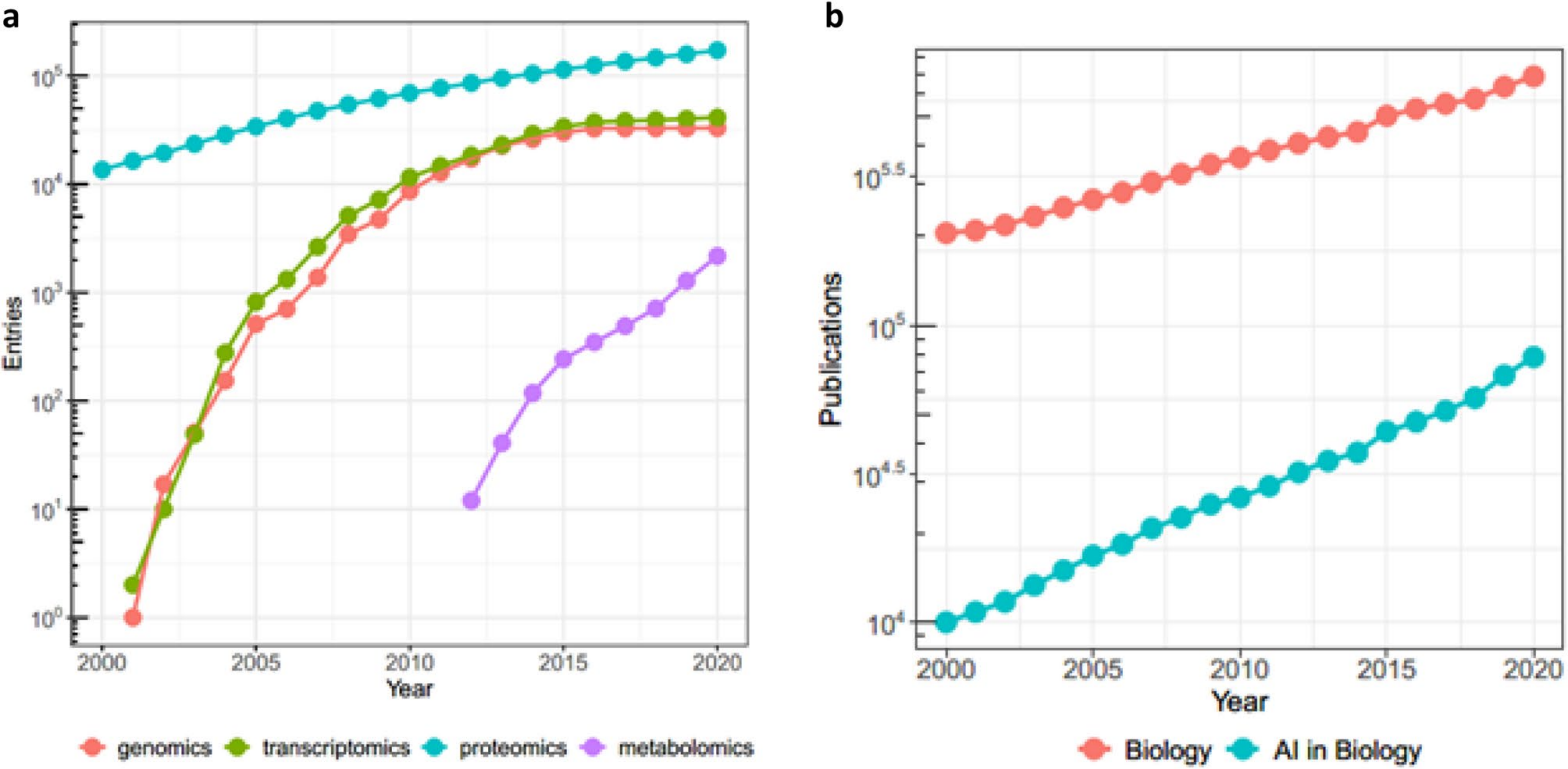
Data growth and publication trends in biology and AI. (**a**) Growth of data in biology: increase in number of entries in 3 main databases from four areas of biology: genomics, transcriptomics (ArrayExpress), proteomics (Protein Data Bank), and metabolomic (MetaboLight). (**b**) Number of publications in Biology and Artificial Intelligence in Biology per year. Data were retrieved from the Web of Science Core Collection as described in the Supplementary Materials.

The unprecedented advance in technology over the past decades and the steep decline in costs have led to a surge in data production from different fields that is still ongoing. Astronomy, high-energy physics, and the health domain, where the rate of data growth is exceeding any other fields^1^, are now all rightfully in the realm of ‘big data’^2^ (Fig. 1a), a term used to define sets of information that are too large or complex to handle with standard methods^3^. This massive growth in data production, together with the creation of public repositories (examples in the health domain are Gene Expression Omnibus, ArrayExpress, Genomic Data Commons) where data can be stored and shared with the scientific community, has posed researchers an unprecedented opportunity for data analysis, but also new challenges derived by the complexity, heterogeneity and high dimensionality of the available information. Whereas standard techniques are often not applicable, AI methodologies have obtained promising outcomes in processing scientific data, resulting in an almost eightfold increase in AI publications in biology only since 2000 (Fig. 1b). However, this growth of AI applications has perplexingly not seen a comparable advance in our understanding of the field, mainly due to the low reproducibility of the published results and a poor generalisation of the findings.

The ability of independent researchers to reproduce a study’s results is key to scientific progress. Nevertheless, the existence of a “replication crisis” is now widely accepted in several fields, such as health, where it has been highlighted in high impact papers^4–6^, natural sciences^7^, and generally STEM (Science, Technology, Engineering, and Mathematics)^8,9^. The reasons behind this lack of reproducibility are many: commonly found limitations include the absence of the actual computer code and dataset used for the analysis, missing description of crucial details of the adopted methodologies, and incomplete reporting of findings, as highlighted very clearly in recent literature^10–16^. Moreover, modern AI is rather diverse, and there are many areas where progress is driven by empirical (and lab-dependent) approaches that often perform very well in specific cases but have no theoretical justification. Although reproducibility is challenging in such cases, we argue that empirical approaches should nevertheless be transparent and traceable.

In addition to, and linked with, the replication challenge, published studies frequently report over-optimistic results that fail the generalisation requirement to allow for reliable employment of AI models to new data. Biased results are often caused by misapplication of AI techniques and study design, such as misuse of the training/test sets during the development and assessment of the algorithm. A standard approach for model derivation and evaluation consists of splitting the initial data into training and test sets; the first is used to train a model, and the second to test its performance. However, this procedure is strongly dependent on the selection of the split (in terms of both set sizes and assignment of samples to the training or test set) and thus can produce highly unstable results^17^. Resampling is a common methodology adopted to estimate the performance of a machine learning model by using randomly drawn subsets of the initial data for training and testing. Nevertheless, the training sample size is commonly fixed and not regarded as an element of variability that should be considered in the algorithm performance report. Another common cause of bias is the incorrect application of an initial pre-processing step. For example, the input matrix is often pre-processed to reduce the data dimensionality (and thus, often, the computation time) and focus on the *input variables* (also called *features*, *predictors*, *covariates*, *explanatory variables*), showing some desired statistical properties. However, this features screening strategy is frequently implemented in a *supervised* manner by looking for the predictors that show a good correlation with the response variable. The problem with this approach is that the selected features have an unfair advantage as they are chosen based on the entire set of observations. Therefore, the estimation of the performance of the learning methodology on the test set is biased since the explanatory variables have already seen the left-out data^18^.

The importance of model training and testing is even greater if we consider that modern ML models often lack explainability, that is, model decisions and/or output cannot be easily or immediately understood by humans. In this scenario, the testability of a model determines its usefulness, and providing rigorous protocols for model training/testing is crucial. The scientific community is then demanding the adoption of (a) standardised pipelines providing robust and reproducible analysis and (b) accessible and usable reports to reduce research waste.

As a result of these needs, several tools have been developed to facilitate the process of building AI models, including the popular scikit-learn (https://scikit-learn.org), classification and regression training, aka caret (https://rdrr.io/cran/caret), TensorFlow (https://www.tensorflow.org), Keras (https://keras.io), and PyTorch (https://pytorch.org). However, these libraries require extensive computer coding and machine learning experience, making them accessible only to scientists with specific theoretical knowledge and programming skills. Software offering guidance in model building and graphics interfaces has recently been developed, for example, ELKI (https://elki-project.github.io), WEKA (http://old-www.cms.waikato.ac.nz/ml/weka), Orange (https://orangedatamining.com), KNIME (https://www.knime.com/knime-analytics-platform). While offering multiple options for applying AI methodologies, these tools do not usually provide an exhaustive and automated pipeline to prevent over-optimistic estimates. The result is that users are responsible for correctly developing a generalisable model, a nontrivial task that requires both mathematical and programming numeracy, and also extensive knowledge of the AI domain. A recent attempt to overcome these issues is represented by Sequential Iterative Modeling “Over Night”, aka SIMON^19^ (https://genular.org): based on caret, it introduces a simplified setup of the analysis and a unified process of the robust model building and evaluation on unseen data. While representing an advancement, SIMON also has limitations, such as the shortage of sampling methodologies, which are limited to cross-validation. Furthermore, the existing solutions lack a generalised automated creation of transparent reports that would facilitate both the assessment of the quality and reliability of derived models, thus disfavouring reproducibility and, subsequently, their adoption in wider practice.

Interesting frameworks aiming to automate the model-building process, such as Automated Machine Learning^20^ (AutoML) and Combined Algorithm Selection and Hyperparameter Optimization^21^ (CASH), have also been proposed. While these are excellent initiatives, particularly suited for mature ML problems near to the final application, they may be less suitable for the research phase, where active user involvement is crucial for inspecting and selecting feature screening methods, hyperparameters, and performance metrics during model building and training.

It is important to highlight that not only new tools but also new modalities have been suggested to address the replication crisis. For instance, in STEM and medical contexts, competitions like DREAM Challenges (iscb.org) have been organized. In these contests, model building and testing occur on the hardware and software systems of the organizers. In such competitions, the code is typically deposited and evaluated on identical and unseen datasets, thus ensuring robust testing and reproducibility. However, while competitions offer a rigorous testing ground, they often pose complex organizational challenges and can be costly. As a result, they may not provide a universally applicable solution for robust and reproducible machine learning in everyday STEM research.

Finally, the AI community has recently proposed guidelines and checklists, e.g. DOME^22^ (Data, Optimization, Model and Evaluation), MI-CLAIM^12^ (Minimum Information about CLinical Artificial Intelligence Modeling), MINIMAR^23^ (MINimum Information for Medical AI Reporting), which should be considered and integrated as part of the process and are currently overseen.

Aiming to address these open challenges, we present RENOIR (REpeated sampliNg methOds for machIne leaRning) a scalable modular open-source software for the standardised development and application of machine learning models. RENOIR provides a comprehensive set of tools and features to facilitate robust model development and enhances the quality of studies using AI by boosting reproducibility and generalisation of findings, thus reducing research waste.

Elements of novelty introduced by RENOIR include the evaluation of the performance of a learning methodology considering sample size dependence through a multiple sampling approach, computation of feature importance scores derived from repeated sampling, ready-to-use filtering functions for supervised feature screening in biomedical analysis, and automated generation of interactive reports following community guidelines for transparency and exploratory analysis.

We illustrated the use and capability of RENOIR by firstly applying it to benchmark datasets (two health datasets, one computer science dataset, and one STEM dataset) and replicating previously reported results. Then, we summarised its successful application in two recently published cancer studies, where we focused on the identification of classifiers for SET2D and TP53 mutation status. Finally, we used RENOIR to address a major pharmacological challenge as a use case.

Source code, documentation, and tutorials demonstrating how to use RENOIR for classification and regression problems, including the examples and the use case reported here, are available on GitHub.

## Results

### RENOIR workflow

**Figure 2:**
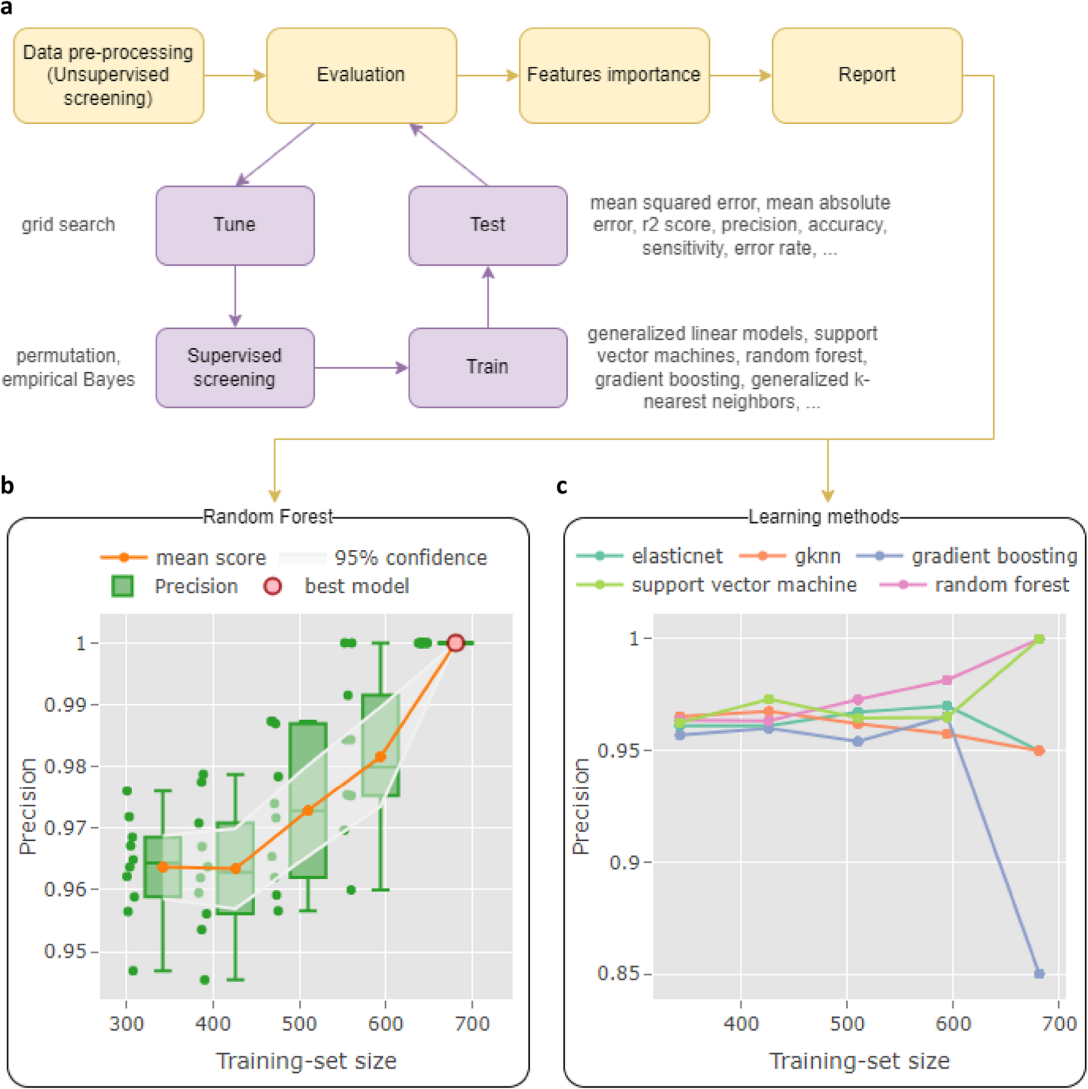
Analytical framework for robust machine learning analysis in biology: simplified RENOIR workflow. (**a**) The main steps can be summarised by an initial pre-processing, the evaluation of the learning method, the computation of the importance of the features, and the creation of the report. During the evaluation, models are built using a nested sampling approach. (**b**) Evaluation of random forest technique in distinguishing benign vs malignant breast cancer samples from the University of Wisconsin Hospitals. Precision values for models built across 5 training-set sizes (342, 426, 510, 594, 681) are shown as dots, while their distribution is illustrated as box plots. The uncertainty of the performance estimation was computed as 95% confidence interval and is represented as bands around the mean value. The automatically selected overall best model (see “Methods” for further details) is highlighted in red. (**c**) Evaluation of five learning methods, including the random forest, using RENOIR in the same breast cancer scenario as (**b**). For each technique, the estimated mean performance metric on the test set, at a given training-set size, is reported as a dot.

RENOIR workflow (Fig. 2a) comprises four main steps: (i) an initial optional pre-processing step, where the feature space dimension is reduced through unsupervised feature selection; (ii) the evaluation of the learning method, wherein the chosen technique is used to fit the models and assess them on the left-out data using a multiple resampling approach; (iii) the computation of the feature importance scores based on the evaluation step; and (iv) the creation of the interactive report.

The adoption of a uniform interface across different ML techniques, as in the popular above-mentioned tools, facilitates usage and reduces learning time by hiding method-specific syntaxes, thus making RENOIR accessible even to individuals with basic coding skills. Renoir avoids over-optimistic performance estimation of a learning methodology by implementing a pipeline for a robust evaluation of the generalisation error through the employment of a nested repeated sampling approach spanning multiple training set sizes. This is an element of novelty with respect to popular existing tools, providing important information on the dependence of the performance of the algorithm on the training sample size. Moreover, global feature importance is calculated from all the evaluated models, ideally producing more robust scores than those computed with common approaches using unique sample sizes. The quantification of the uncertainty related to the performance estimate is included in the process, thus providing measures of expected variability for the computed results. Appropriate *unsupervised* pre-processing strategies for initial dimensionality reduction are integrated to facilitate the correct application as part of the process. Moreover, RENOIR implements an optional *supervised* feature screening stage incorporated in the learning steps, that is, before the training of the model but after the data are sampled into independent training/validation/test sets. Finally, to improve interpretation and ensure reproducibility, RENOIR facilitates automated creation of interactive reports following community recommendations. In particular, the reports contain not only a summary of the adopted methodologies (for example, AI algorithm, sampling strategy, and performance metrics) but also an overview of the performed evaluation in tabular and graphical forms, thus allowing an immediate inspection of the findings.

### Methodology and model evaluation visualisation

Two types of reports can be generated, one for the evaluation of the learning methodology and the other for the evaluation of a specific model:

- The report for the learning methodology contains the main information of the adopted approach and an overview of the performed evaluation; information on the selected learning method, data pre-processing (unsupervised feature screening), sampling strategy, hyperparameter optimisation, supervised feature screening, overall best model selection, and feature importance are outlined here. Moreover, an overview of the performance of all the built models across the training-set sizes over the training, testing and full data is provided in tabular and graphical form (Fig. 2b).
- The report for the model serves as a container for model-specific evaluation metrics. It includes the performance of the model over the internal and (if provided) external data in tabular and graphical forms. For example, the report of classification problems includes the confusion matrix, performance metrics for each target class and average scores, receiver operating characteristic (ROC) curve, principal component analysis (PCA) plot showing the variability of the data (where only the features of the model are considered) over the first principal components, and violin plots showing the distributions of summarised features across classes.

### RENOIR application to benchmark and biomedical datasets

To evaluate our selection of AI techniques and demonstrate the applicability of RENOIR to the health and STEM domains, we used different datasets for classification and regression problems from the UC Irvine Machine Learning Repository website. These include two health datasets (breast cancer data from the University of Wisconsin Hospitals and heart-disease data from the Cleveland Clinic Foundation), one computer science dataset (relative CPU Performance data from Ein-Dor and Feldmesser, 1987), and one STEM dataset (city-cycle fuel consumption data from the Carnegie Mellon University Statistics library). The obtained mean performance metrics were comparable with previously reported results in the benchmark repository, validating the RENOIR integration of the chosen ML methodologies (see Supplementary Information).

To demonstrate the additional functionalities of RENOIR, including a direct comparison between algorithms, model evaluation, and transparent reporting, we decided to focus on the evaluation of different learning methods applied to one of these well-known classification problems.

In particular, we investigated the diagnosis of 683 breast cancer samples (444 benign and 239 malignant) obtained from the University of Wisconsin Hospitals by using five approaches: generalized linear models with penalization (elasticnet), random forest, support vector machine (SVM) with linear kernel, gradient boosting models (gbm), and generalized kNN (gknn). Nine cytological characteristics commonly described as distinguishing between benign and malignant breast fine-needle aspirates (FNAs) were assessed on a scale of 1 to 10 (10 being the closest to malignant) and used as a set of predictors: single epithelial cell size, uniformity of epithelial cell size and of cell shape, normal nucleoli, clump thickness, marginal adhesion, bare nuclei, blandness of nuclear chromatin, and infrequent mitoses. Given the small number of input variables, unsupervised or the supervised screening steps were not applied. The samples were repeatedly split into train/test sets at five different training set sizes (342, 426, 510, 594, and 681) via random sampling, and the test set was used to assess the goodness of the fitted model. The training sets were furtherly divided into train and validation sets via tenfold cross-validation to tune the hyperparameters of the models.

The outcome of the evaluation of the learning methods on the test sets is shown in Fig. 2c, where the estimation of the mean performance is reported for each considered training set size. Interestingly, the random forest technique seemed to perform better than the others for most of the training sizes, reaching the maximum precision (similar to the SVM) for the leave-two-out case, where most of the data were used for training and only two samples were left in the test set.

The precision of the different random forest models built across the five training set sizes is reported in Fig. 2b. In this plot, the estimation of the mean performance metric (orange line) is enriched by the metric value for each model (green dots), their distribution in the form of boxplots, and quantification of the uncertainty of the estimation as 95% confidence intervals (transparent white bands around the mean score).

To further emphasise the relevance of RENOIR to biomedical science, we highlight two recently published applications^24,25^.

In our first investigation, we explored the functional impact of SETD2 loss and its association with DNA methylation changes across diverse cancer types. RENOIR played a pivotal role in the development of a robust model for diagnosing SETD2-mutated renal cancers. Particularly, we used unsupervised and supervised feature screening followed by generalized linear model selection with L1/L2 penalisation, with hyperparameters optimised in tenfold cross-validation. Four training set sizes (20, 46, 72, 98) were considered, and the overall best model was selected based on the minimum test error across all models fitted using the training set size showing the lowest upper bound of the 95% CI. The methylation data, obtained from the landmark cancer genomics program The Cancer Genome Atlas (TCGA), encompassed 309 samples and 384,560 CpG sites. RENOIR’s contribution enabled the identification of a 3-CpG model with an 89% accuracy in correctly classifying renal cancer samples as SETD2 cases or wild type (WT). Notably, the identified model was further validated in an external cohort of renal cancer patients, demonstrating a classification accuracy of 87%^24^.

In another application, we focused on unravelling the functional consequences of TP53 mutations, the most frequently mutated gene in human cancers. Here, we harnessed the power of RENOIR to develop classifiers predicting the mutation status of TP53 from transcriptomic data, sourced from two well-established databases—the Cancer Cell Line Encyclopedia (CCLE) and The Cancer Genome Atlas. Our study spanned 1457 cell lines across 22 cancer types in CCLE and 12,531 cancer samples across 54 cancer types and subtypes in TCGA. Two distinct feature spaces were considered: one comprised of four genes previously identified for their significant correlation with TP53 mutation in clinical samples, and the other consisting of a set of target genes constituting the TP53 regulatory network, also known as the regulon. Similar to the previously mentioned study, we fitted generalized linear models with L1/L2 penalisation using different training set sizes, with hyperparameters optimised in tenfold cross-validation. Notably, the identified best models demonstrated their efficacy in predicting TP53 mutation status (wild type/mutant) based on the expression of the TP53 regulon^25^.

### Use case

**Figure 3:**
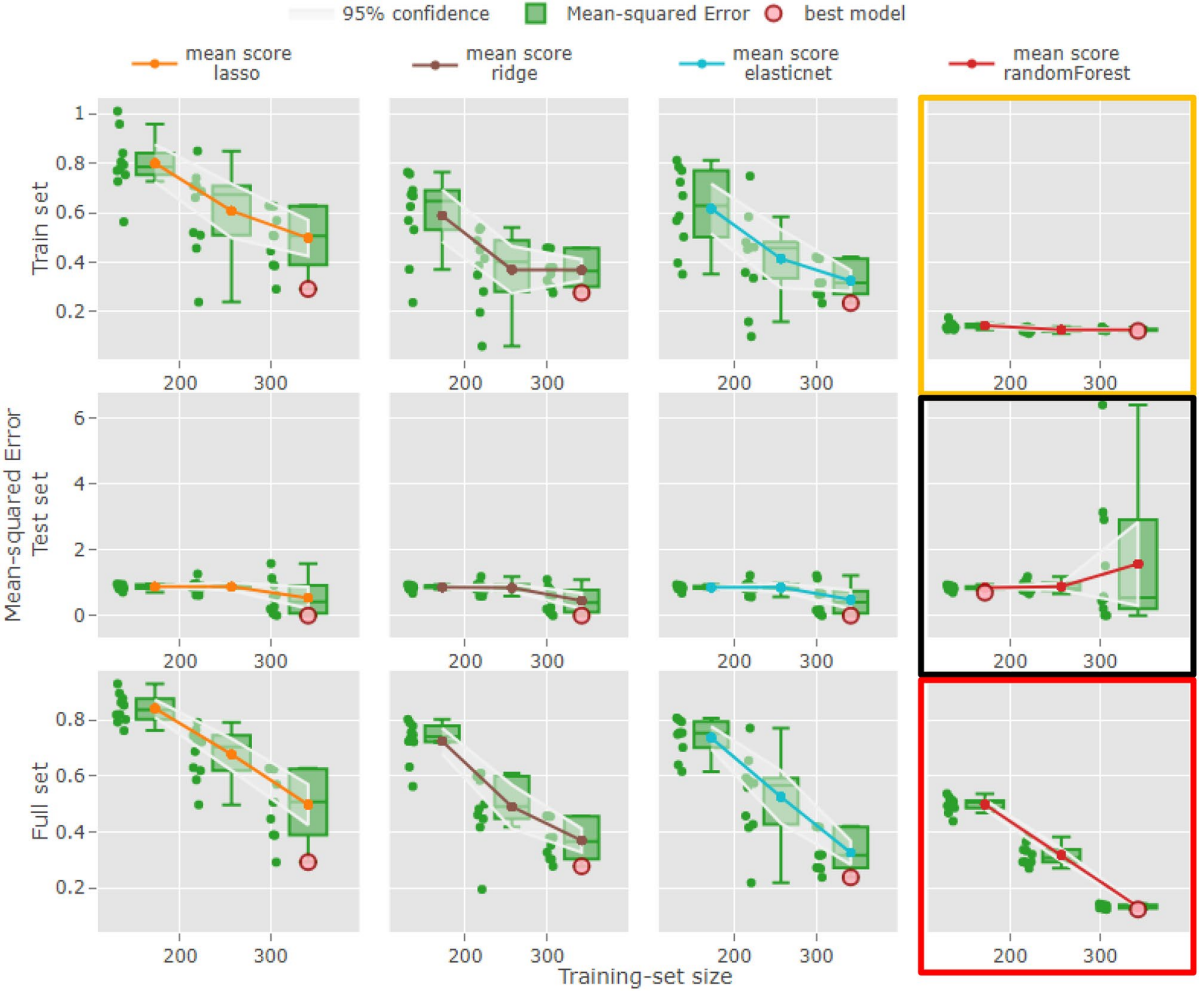
Application of RENOIR to drug response data for cancer cell lines: comparison of models’ performance. Four different methodologies (i.e., lasso, ridge, elasticnet, random forest) were used to train a model able to predict IC50 values from genomic features. Three training set sizes (172, 257, 342) were considered. The performance of the models computed on the train, test, and full sets is here reported as mean-squared error for estimating the IC50 and depicted as green dots. For each technique, the mean-squared error of the different models built across the 3 training set sizes is reported in the figure, while their distribution is illustrated as box plots. The uncertainty of the performance estimation was computed as 95% confidence interval and is reported on the figure as bands around the mean value. The automatically selected overall best model (see “Methods” for further details) is highlighted in red. The learning method showing the best performance during training is random forest (indicated by a yellow box), having an error constantly between 0 and 0.2 across the different training set sizes. However, random forest performs similarly to the other techniques when assessed on left-out data (black box), revealing a worse behaviour (in terms of both mean error and CI) when comparing the models computed using a training set size of 342.

After benchmarking RENOIR over commonly used datasets and reporting its successful application in recent studies focusing on the identification of classifiers for SET2D mutation status in renal cancer and TP53 mutation status in cancer cell lines and samples, we employed our software to address a major pharmacological challenge: the prediction of drug efficacy^26^. In particular, we considered a pharmacogenomics dataset of cancer cell lines produced as part of the Genomics of Drug Sensitivity in Cancer (GDSC) project, where more than 1000 human cancer cell lines, each with gene expression characterized for more than ten thousand genes, were screened with a wide range of anti-cancer drugs to study the molecular features that influence drug response. Reported measures of drug sensitivity include the half maximal inhibitory concentration (IC50) and slope of the dose–response curve. We decided to focus on predicting the IC50 of the drug bortezomib by using these genes as predictor variables. Bortezomib is a treatment for myeloma and mantle cell lymphoma, and data from a phase II/III clinical trial in patients with relapsed multiple myeloma are available for independent validation (see Supplementary Material). First, we removed the cell lines without bortezomib response information and selected the top 80% variable genes by using the median absolute deviation as a metric of variability, resulting in a dataset of 343 cell lines (i.e., the observations) and 9432 genes (i.e., the features). Then, we developed different models using the lasso, ridge, elasticnet, and random forest techniques (Fig. 3). This allowed us not only to derive ML models for prediction of drug efficacy based on the gene expression outlook of a cell, as previously done^26^, but also to enable full inspection of the models, so that the performance achieved by different methodologies can be compared while assessing the generalizability of the models by varying the size of the train-test sets.

The performance of the models (calculated as the mean-squared error for estimating the IC50) on the training, testing, and full sets is reported in Fig. 3. The green dots represent the mean-squared error values computed by the tuned models resulted from fivefold cross-validation, and the boxplots their distributions. This approach provides an immediate and clear estimate of the variability of the chosen performance metric, that is, the mean-squared error. The mean resampling metrics and 95% confidence intervals are also shown in the plots. Looking at the figure, we can see that the learning method with the best performance during training across different training set sizes is the random forest, which shows an average resampling error between 0.15 and 0.1 (yellow box), while lasso is between 0.8 and 0.45, ridge between 0.6 and 0.35, and elasticnet between 0.65 and 0.3. Focusing on the performance on the test set, the random forest performed similarly to the other techniques when assessed on left-out data (black box), revealing a worse behaviour (in terms of both mean error and CI) when comparing the models developed using a training set size of 342. Looking at the full set, the random forest again showed the best performance at each training set size (red box). Overall, the random forest seems to be the learning method with the best performance on the training and full sets. However, the deterioration of the performance on the test set of all learning methods (second row in the figure, average resampling scores between 0.45 and 1.6) might indicate that the developed models could be inadequate for the prediction of external data.

## Conclusion

AI techniques are revolutionising our capability to analyse complex and large scientific datasets. However, lack of reproducibility and poor generalisation are two significant challenges affecting AI publications. In response to these critical issues, we developed RENOIR, a software implementation of a generalized analytical framework using resampling for training and testing, ensuring robust and reproducible machine learning model development.

RENOIR adopts standardised pipelines and automated generation of transparent reports, enhancing reproducibility. Its object-oriented implementation supports straightforward expansion of currently supported settings. Moreover, clear interactive plots and summary tables integrated in the reports facilitate exploratory analysis and make our software accessible to a broader audience, beyond computer science and bioinformatics experts.

To demonstrate RENOIR’s versatility, we applied it to benchmark datasets across biomedical, computer science, and STEM domains, reproducing previous results. Two successful applications of RENOIR to cancer research focused on studying the functional impact of SETD2 loss and TP53 mutations. Notably, our tool identified a robust model for diagnosing SETD2-mutated renal cancers, exhibiting strong generalisation in an independent cohort. Furthermore, we showcased RENOIR’s capacity to evaluate different learning methods in predicting drug efficacy.

Overall, our analyses underlined RENOIR’s versatility and its ability to facilitate the identification of robust and reproducible models, thus making RENOIR a valuable tool for developing machine learning models across diverse domains.

## Methods

### Evaluation

#### Learning method evaluation

The main idea behind RENOIR is to estimate the mean performance of a learning methodology via a multiple random-sampling approaches spanning different training set sizes. For each considered size, data is randomly split into independent training and test sets multiple times, the training set used to build the models, and the left-out set to assess the performance. The computed measures of assessment are then used to obtain an estimate of the mean performance and the related 95% confidence interval.

#### Multiple random sampling

The training set size is often fixed in model development and validation. For example, a popular approach is given by the $k$-fold cross-validation, where the input data is randomly split into $k$ groups, with $k$ ranging from 2 to $n-1$ ($n$ being the number of available observations): then, for $k$ times, one group is used to test the goodness of the model developed using the combined remaining $(k-1)$ folds. In such scenario, a commonly used selection is $k=10$. However, there is no specific rationale for this choice.

While it is known that generally the variance of the model performance on the test set is increasing with $k$ (and therefore with the training set size), the identification of the best value of $k$ ensuring optimal evaluation of model validity is a current topic of study in the literature^27^.

RENOIR allows the exploration of the impact of the randomization process and training set size over the model performance, thus informing the user on the stability of the chosen algorithm.

#### Mean performance

An estimate of the mean performance for the $i$-th training set size is computed as the sample mean:

$${M}_{i}={\widehat{\mu }}_{i}={sample\, mean}_{i}=\frac{1}{n}\sum_{j=1}^{n}{performance}_{ij},$$

where ${performance}_{ij}$ is the average performance of the $j$-th model (see “Performance measures” for further information), and *n* is the number of repeated samples taken (and, therefore, models computed) at the considered training set size. Note that *n* is kept constant across training set sizes.

#### Uncertainty quantification

To quantify the uncertainty related to the computed mean performance, we calculate the so-called confidence interval (CI). The CI is a range of values providing additional information about the variability of the point estimate. The CI of the mean performance for the $i$-th training set size is defined as:

$${CI}_{i}={estimate}_{i}\pm {margin\, of\, error}_{i}={estimate}_{i}\pm critical\, value\times {standard\, error\, of\, the\, sample\, mean}_{i}={M}_{i}\pm c\times {SEM}_{i,}$$

where the $critical \,value$ depends on the sampling distribution of the estimate and the desired confidence level (our default value is 95% confidence level), and the standard error of the mean estimate (${SEM}_{i}$) is the standard deviation of the sample mean and is computed as:

$${SEM}_{i}=\frac{{standard\, deviation\, of\, performances}_{i}}{\sqrt{n}}=\frac{{s}_{i}}{\sqrt{n}}.$$

The sample standard deviation of the performance metrics (${s}_{i}$) is calculated as the squared root of the sample variance using the unbiased estimator:

$${Var}_{i}={s}_{i}^{2}=\frac{1}{n-1}\sum_{j=1}^{n}{({performance}_{ij}-{M}_{i})}^{2}.$$

So, the ${SEM}_{i}$ is:

$${SEM}_{i}=\sqrt{\frac{1}{n(n-1)}\sum_{j=1}^{n}{({performance}_{ij}-{M}_{i})}^{2}.}$$

#### Best model selection

RENOIR automatically selects an overall best model for each considered performance measure by firstly choosing the optimal training set size, i.e., the size showing the lowest upper (or highest lower) bound of the 95% CI. Then, it selects the model with the optimal (i.e., minimal or maximal) score across all the trained models built using such size.

#### Model evaluation

While the evaluation of the learning method is performed on the provided dataset randomly split into training/test sets multiple times, RENOIR also allows for the quick evaluation of the single model on an external dataset furtherly provided by the user.

### Performance measures

Several performance metrics are available for classification and regression problems, including misclassification error, accuracy, precision, sensitivity, F1 score, mean-squared error, root-mean-square error, mean absolute error, mean absolute percentage error, r2 score.

#### Classification

In the formulas below P (Positive) and N (Negative) represent the actual condition; PP (Predicted Positive) and PN (Predicted Negative) represents the predicted conditions; TP (True Positive) is the number of Positive conditions correctly predicted, while FN (False Negative) is the number of Positive conditions wrongly predicted as negative (P = TP + FN); FP (False Positive) is the number of Negative conditions wrongly predicted as Positive, while TN (True Negative) is the number of Negative conditions correctly predicted (N = FP + TN). An immediate representation of this information is represented by the *table of confusion* (also called *confusion matrix*).

**Table:** 

		Predicted condition	
		PP (predicted positive)	PN (predicted negative)
Actual condition	P (positive)	TP (true positive)	FN (false negative)
	N (negative)	FP (false positive)	TN (true negative)

#### Misclassification error (classification error rate)

The classification error rate measures the fraction of all instances that are wrongly categorized. It is defined as:

$$ERR=\frac{errors}{total}=\frac{FP+FN}{P+N}=\frac{FP+FN}{TP+FP+TN+FN}=1-ACC.$$

#### Accuracy

The accuracy is the complement of the classification error, and it measures the fraction of all instances that are correctly categorized. It is defined as:

$$ACC=\frac{correct}{total}=\frac{TP+TN}{P+N}=\frac{TP+TN}{TP+FP+TN+FN}=1-ERR.$$

#### Precision

The precision is the fraction of correctly predicted elements within a condition. It is defined as:

$$precision=positive\, predictive\, value\, (PPV)=\frac{TP}{PP}=\frac{TP}{TP+FP}=1-FDR.$$

#### Sensitivity

The sensitivity measures the ability of a classifier of correctly predicting the presence of a condition. It is defined as:

$$sensitivity=recall=true\, positive\, rate\, (TPR)=\frac{TP}{P}=\frac{TP}{TP+FN}=1-FNR.$$

#### F1 score

The traditional F1 score is a measure of accuracy and is computed as the harmonic mean of precision and sensitivity:

$$F1=2\frac{precision\times sensitivity}{precision+sensitivity}=\frac{TP}{TP+\frac{1}{2}(FP+FN)}.$$

#### Regression

In the following formulas, $y$ is the true value, ${y}_{i}$ is the true value of the $i$-th sample, $\widehat{y}$ represents the predicted value, ${\widehat{y}}_{i}$ is the predicted value of the $i$-th sample, $n$ is the number of samples.

#### Mean-squared error

The mean squared error (MSE) is a measure of errors based on squared losses. The MSE estimated over $n$ observations is defined as:

$$MSE(y,\widehat{y})=\frac{1}{n}\sum_{i=1}^{n}{({y}_{i}-{\widehat{y}}_{i})}^{2}.$$

#### Root-mean-square error

The root-mean-square error (RMSE) is a measure of errors based on squared losses. It is defined as the squared root of the MSE:

$$RMSE(y,\widehat{y})=\sqrt[2]{MSE(y,\widehat{y})}=\sqrt[2]{\frac{1}{n}\sum_{i=1}^{n}{({y}_{i}-{\widehat{y}}_{i})}^{2}.}$$

#### Mean absolute error

The mean absolute error (MAE) is a measure of errors based on absolute values. The MAE estimated over $n$ observations is defined as:

$$MAE(y,\widehat{y})=\frac{1}{n}\sum_{i=1}^{n}|{y}_{i}-{\widehat{y}}_{i}|.$$

#### Mean absolute percentage error

The mean absolute percentage error (MAPE) is a measure of errors based on relative absolute values. The MAPE estimated over $n$ observations is defined as:

$$MAPE(y,\widehat{y})=\frac{1}{n}\sum_{i=1}^{n}\frac{|{y}_{i}-{\widehat{y}}_{i}|}{\underset{}{{\text{max}}}(|{y}_{i}|,\varepsilon )},$$

where $\varepsilon$ is an arbitrary positive small number used to avoid a division by zero.

#### R2 score

The coefficient of determination (R squared) is a measure of the proportion of variance in the dependent variable ($y$) that is explained by the independent variables. The R2 estimated over $n$ observations is defined as:

$${R}^{2}(y,\widehat{y})=1-\frac{\sum_{i=1}^{n}{({y}_{i}-{\widehat{y}}_{i})}^{2}}{\sum_{i=1}^{n}{({y}_{i}-\overline{y})}^{2}},$$

where $$\overline{y}=\frac{1}{n}\sum_{i=1}^{n}{y}_{i}$$.

### Learning methods

Renoir currently supports several methods for regression and classification problems, including generalized linear models with L1/L2 penalisation (lasso, ridge, elastic-net), random forests, support vector machines, boosted models, generalized k-nearest neighbours.

### Data pre-processing (unsupervised screening)

To facilitate the correct application of an initial *unsupervised* screening, RENOIR offers an integrated data filtering step, where features are selected on the application of three possible rules:

(i) Missing value ratio: variables with a number of missing elements exceeding a specified cutoff are removed.
(ii) Threshold-based selection: features with values below a certain threshold are filtered out. Typically applied to gene expression data, this rule is motivated by the notion that a gene must exhibit a minimal level of expression to be biologically relevant. A commonly used cut-off is the median expression of genes in a sample. Further refinement includes retaining features only if they surpass the threshold in a specified number of samples.
(iii) Variability-based selection: prioritizing features with higher variability across samples, this rule aims to capture interesting variations linked to experimental conditions, such as drug administration. Variability can be measured using standard deviation, interquartile range, or median absolute deviation.

### Resampling

RENOIR supports three types of methods to randomly split the input data into independent training and evaluation sets: sampling without replacement, sampling with replacement (bootstrap), and *k*-fold cross-validation. If a stratification variable is provided, a stratified approach is adopted, where the proportion of the strata in the full observation set is tried to be maintained in the samples (*proportionate allocation*). A balanced strategy, where the strata frequency in the samples is tried to be balanced, can also be selected for the sampling with and without replacement.

### Hyperparameters optimisation

When the considered learning methodology has hyperparameters (i.e., parameters used to control the learning process that should be fixed before the training), RENOIR allows for their optimal selection via a traditional *grid search*. In this approach, a provided subset of the hyperparameter space is searched to identify the set, also called *configuration*, that achieves the optimal value for a chosen performance measure. This procedure is also referred to as *tuning* of the model. During the tuning, the randomly selected training set is furtherly randomly split into multiple training and validation sets by using one of the methodologies described above. Each configuration will have an associated mean score computed over the measures calculated on the different validation sets, and the one resulting in optimal (i.e., maximal or minimal, depending on the metric) mean value is chosen as the “best” configuration. Given that selecting optimal hyperparameters could generate models which overfit^28^, RENOIR also selects a second configuration by following the “*one standard error*” (1SE) rule, such that the mean resampled error is within one standard error of the optimum. The idea is to choose the simplest model whose performance is comparable with the best model, potentially achieving better generalisation.

Once the hyperparameters are fixed, the model is trained on the entire training set.

While RENOIR currently supports only grid search—a basic yet sometimes computationally expensive approach—we plan to expand the methods for hyperparameter optimization to encompass a broader range of strategies.

### Features screening (supervised screening)

Computational cost, model interpretability, and estimation accuracy are three main concerns in ultra-high dimensional data settings, where the number of variables is much larger than sample size. Genome-wide association studies (GWAS) are a common example of such settings, where hundreds of thousands of genetic variants are screened to look for associations with a trait of interest. In this context, the chance of finding unimportant variables highly correlated with important ones increases with dimensionality, thus increasing the noise and making harder to discriminate between them^29^. It is well-known that the classification in high-dimensional settings suffers from the noise features that do not contribute to the reduction of the misclassification rate up to the point that some methods cannot perform any better than random guessing^30^.

A simple and effective idea to approach such problems is to adopt a features screening step before the training of the model, in order to filter out the variables weakly associated with the response. To facilitate the adoption of the methodology, RENOIR implements an optional *supervised* feature screening stage incorporated in the learning steps, i.e., before the training/tuning of the model but after the data is sampled into independent training/validation sets. Two possible options are available for the supervised screening: empirical Bayes moderated t-statistics^31^ test and/or permutation test^32^.

### Features importance

RENOIR tries to compute a score of overall importance for each feature, defined as the weighted mean of the feature importance scores (FIS) across all built models in the entire evaluation procedure. If we consider the i-th feature, then the importance score can be written as:

$${FIS}_{i}=\frac{1}{\sum_{j=1}^{n}{weight}_{j}}\sum_{j=1}^{n}{weight}_{j}\times {importance}_{ij},$$

where $n$ is the total number of models fitted across the different training set sizes, ${weight}_{j}$ stands for the weight associated to the j-th model, and ${importance}_{ij}$ is a numerical value standing for importance of the i-th feature in the j-th model. The weight element is linked to the obtained *performance* measure (see the section “Performance measures” for further information on the available metrics) and is currently computed by squaring the metric in order to give more relevance to the models with good prediction ability (but we plan to extend the available options in the future). If the optimal value of the measures is the minimum, then ${weight}_{j}$ is defined as

$${weight}_{j}=\frac{1}{{({performance}_{j}+\varepsilon )}^{2}},$$

where $\varepsilon$ is a constant added to avoid the division by zero. If the optimal value is obtained by maximization, then

$${weight}_{j}={performance}_{j}^{2}.$$

Finally, ${importance}_{ij}$ is defined by default as

$${importance}_{ij}={presence}_{ij}\times {sign}_{ij}\times {stability}_{ij},$$

where ${presence}_{ij}$ can have binary value 0/1 depending on the presence of the feature in the j-th model; ${sign}_{ij}$ is the sign of the predictor coefficient (where applicable); ${stability}_{ij}$ is an optional numerical value standing for stability of the i-th feature during the tuning of the j-th model, and is computed as the frequency of the feature across all the *m* trained models built during the tuning

$${stability}_{ij}=\frac{1}{m}\sum_{k=1}^{m}{presence}_{ijk}.$$

### Modularity

RENOIR was developed in R programming language following an object-oriented approach where the different aspects of the framework were developed as independent parts of code. The main modules are Renoir (which serves as container for the analysis result), Filter (implementing unsupervised features screening), Evaluator (representing the learning method evaluation flow, i.e. incorporating repeated sampling, tuning, training, testing), Sampler (providing the sampling strategies), Screener (implementing the supervised features screening), Tuner (providing the optimisation of the hyperparameters), Trainer (interface for the model training), Forecaster (dealing with the predictions), Scorer (providing the performance metrics).

The modularity of the package allows for further expansion of our software, for example by adding new learning methodologies, or performance metrics.

### Scalability

The *embarrassingly parallel* nature of the evaluation of a learning method via nested sampling, as well as the hyperparameter optimisation via grid search, allowed for a scalable implementation of these tasks. RENOIR supports the parallel execution through the use of the parallel, doParallel, and foreach R packages.

### Supplementary Information

Supplementary Information.

## Data Availability

RENOIR is currently available in a GitHub repository (https://github.com/alebarberis/renoir). To benchmark the evaluations obtained by RENOIR against external results we retrieved different datasets publicly available from the UC Irvine Machine Learning Repository website (https://archive-beta.ics.uci.edu). The batch-corrected gene expression data for the GDSC cell lines and the clinical trial were retrieved from https://genome.med.nyu.edu/public/tsirigoslab/deep-drug-response (files bortezomib_cells.rds and bortezomib_clinical.rds), a repository linked with the paper from Sakellaropoulos and colleagues^26^. The IC50 scores used for the training of the models were included in the bortezomib_cells.rds file. Patients’ response to Bortezomib was extracted from the supplementary material of the paper (https://ars.els-cdn.com/content/image/1-s2.0-S2211124719314883-mmc2.xlsx).
