# Supplementary material for: Robustness and reproducibility for AI learning in biomedical sciences: RENOIR
Source: Sci Rep. 2024 Jan 22;14:1933. doi: 10.1038/s41598-024-51381-4 (PMC10810363; doi:10.1038/s41598-024-51381-4)
Supplement: Supplementary file 1 — Supplementary Information. [file 41598_2024_51381_MOESM1_ESM.docx]

**Robustness and reproducibility for AI learning in biomedical sciences: RENOIR**

Alessandro Barberis^1,2*^, Hugo Aerts^3,4,5,6^, Francesca Buffa^2,7*^

Institutions:

1. Nuffield Department of Surgical Sciences, Medical Sciences Division, University of Oxford, Old Road Campus Research Building, Roosevelt Drive, Oxford OX3 7DQ
2. MRC Oxford Institute for Radiation Biology, Department of Oncology, Medical Sciences Division, University of Oxford, Old Road Campus Research Building, Roosevelt Drive, Oxford OX3 7DQ
3. Artificial Intelligence in Medicine (AIM) Program, Brigham and Women’s Hospital, Harvard Medical School, Boston, MA, USA
4. Radiation Oncology and Radiology, Dana-Farber Cancer Institute, Brigham and Women’s Hospital, Harvard Medical School, Boston, MA, USA
5. Radiology and Nuclear Medicine, Maastricht University, Maastricht, The Netherlands
6. Cardiovascular Imaging Research Center, Massachusetts General Hospital, Harvard Medical School, Boston, MA, USA
7. Department of Computing Sciences, Bocconi University, and Bocconi Institute for Data Science and Analytics (BIDSA), Milano, Italy

*Co-corresponding authors

**Supplementary material**

**Cost per Human Genome**

The changes in the cost of sequencing a human genome for the past 20 years were retrieved from the National Institutes of Health (NIH) National human Genome Research Institute (NHGRI) website (The Cost of Sequencing a Human Genome).

**Genomics data volume**

We considered as an example of the volume of data in genomics the cumulative number of archived sequenced bases in one of the main important sequencing databases, GenBank. We also considered the cumulative number of entries in the European Bioinformatics Institute (EBI) ArrayExpress database as reported on their website. The search was refined for “Genomics” as “Omics type”.

**Transcriptomics data volume**

We considered as an example of the volume of data in transcriptomics the cumulative number of entries in the European Bioinformatics Institute (EBI) ArrayExpress database as reported on their website. The search was refined for “Transcriptomics” as “Omics type”.

**Metabolomics data volume**

We considered as an example of the volume of data in metabolomics the cumulative number of studies in the EBI MetaboLight database as reported on their website. Data was manually retrieved from the plot.

**Proteomics data volume**

We considered as an example of the volume of data in proteomics the cumulative number of entries in the Research Collaboratory for Structural Bioinformatics (RCSB) Protein Data Bank (PDB) database as reported on their website.

**Number of publications in biology**

The number of publications in biology were retrieved from the Web of Science website using the following query to search the Web of Science Core Collection.

| Query | (TS=( biolog*)) OR (TS=(medic*)) OR (TS=(prote*)) OR (TS=( transcriptom*)) OR (TS=(genom*)) OR (TS=(metabol*)) OR (TS=(multiomic*)) OR (TS=(multi-omic*)) OR (TS=(biomedic*)) |
| --- | --- |
| link | https://www.webofscience.com/wos/woscc/summary/9236ee68-1ed2-4c49-826b-89e2cb11a4cd-14a31316/relevance/1 |

**Number of AI publications in biology**

The number of artificial intelligence publications in biology were retrieved from the Web of Science website using the following query to search the Web of Science Core Collection.

| Query | ((TS=(biolog*)) OR (TS=(medic*)) OR (TS=(prote*)) OR (TS=(transcriptom*)) OR (TS=(genom*)) OR (TS=(metabol*)) OR (TS=(multiomic*)) OR (TS=(multi-omic*)) OR (TS=(biomedic*))) AND ((TS=(machine learning)) OR (TS=(artificial intelligence)) OR (TS=(deep learning)) OR (TS=(supervised learning)) OR (TS=(unsupervised learning)) OR (TS=(reinforcement learning)) OR (TS=(glm)) OR (TS=(generalised linear model)) OR (TS=(support vector machine)) OR (TS=(svm)) OR (TS=(random forest)) OR (TS=(classification)) OR (TS=(regression)) OR (TS=(clustering)) OR (TS=(discriminant analysis)) OR (TS=(nearest neighbor)) OR (TS=(decision tree))  OR (TS=(neural network)) OR (TS=(hidden markov model))) |
| --- | --- |
| link | https://www.webofscience.com/wos/woscc/summary/4a9fefc0-c6df-4d2f-b274-da4e24b0eaed-14a3d443/relevance/1 |

**Benchmark**

To benchmark the evaluations obtained by renoir against external results we retrieved different datasets publicly available from the UC Irvine Machine Learning Repository website (https://archive-beta.ics.uci.edu). In particular, we selected two datasets already used in classification problems:

- The breast cancer data from the University of Wisconsin Hospitals (683 observations, 9 features)
- The heart-disease data from the Cleveland Clinic Foundation (297 observations, 9 features)

And two datasets already used in regression problems:

- The relative CPU Performance data from Ein-Dor and Feldmesser [1987] (209 observations, 6 features)
- The city-cycle fuel consumption data from the Carnegie Mellon University Statistics library (392 observations, 7 features)

**Benchmark classification**

We used the precision reported on the UC Irvine Machine Learning Repository website as reference.

*Breast cancer data from the University of Wisconsin Hospitals*


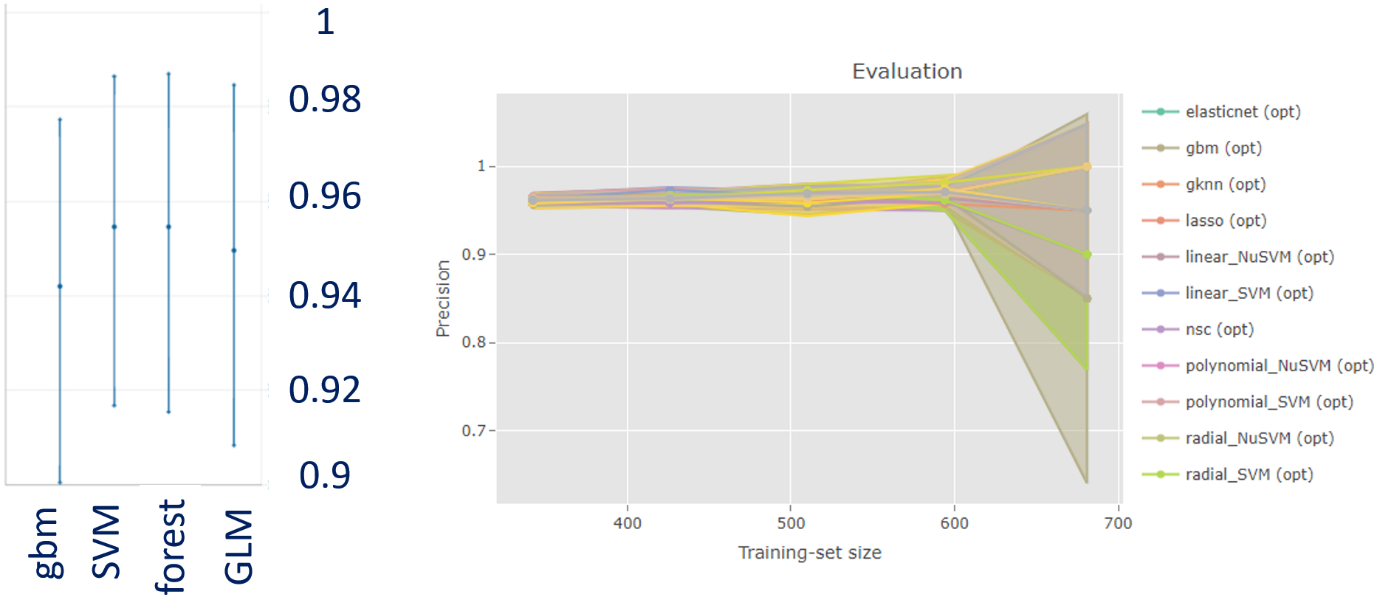


*Heart-disease data from the Cleveland Clinic Foundation*


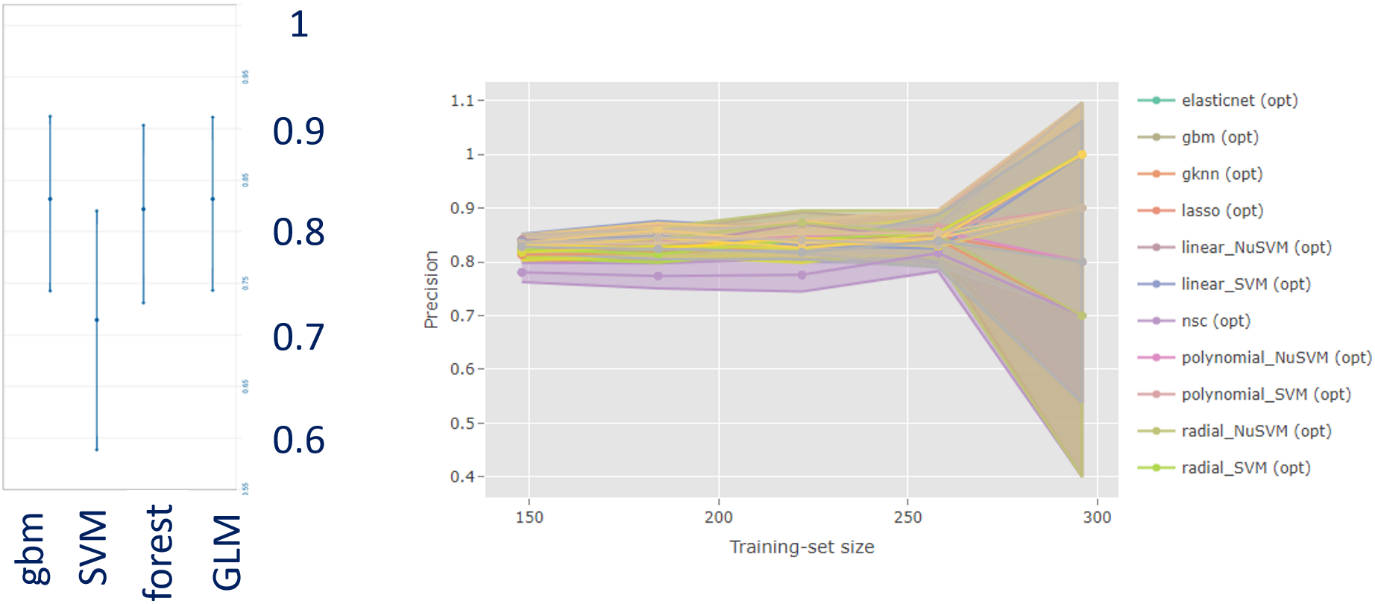


**Benchmark regression**

We used the mean error on unseen data reported in Quinlan (1993) as reference.

*Relative CPU Performance data from Ein-Dor and Feldmesser [1987]*

*
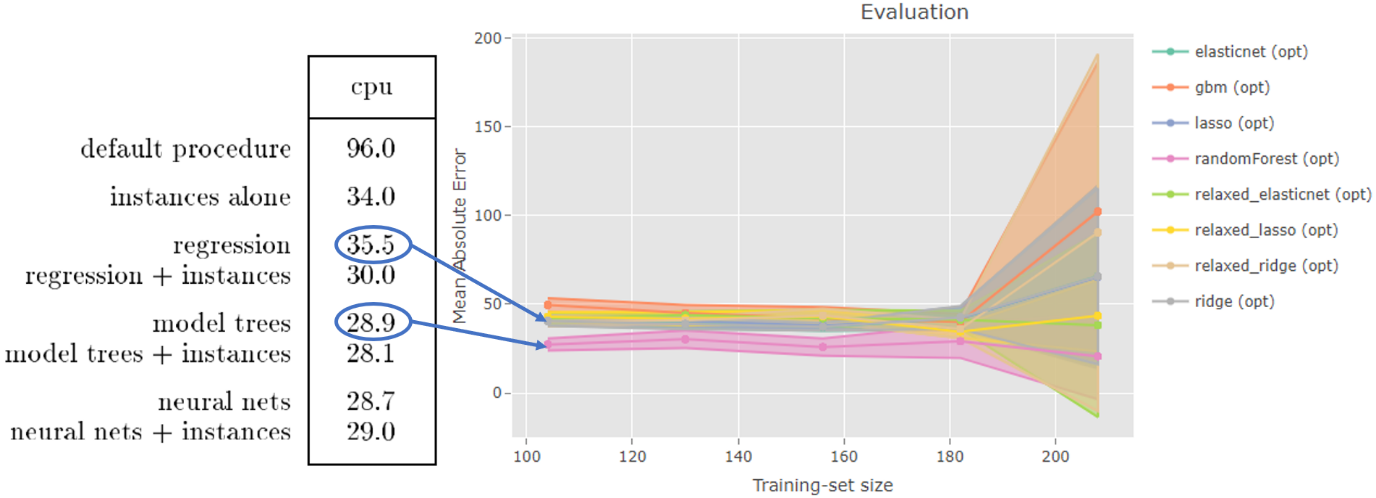
*

*City-cycle fuel consumption data from the Carnegie Mellon University Statistics library*


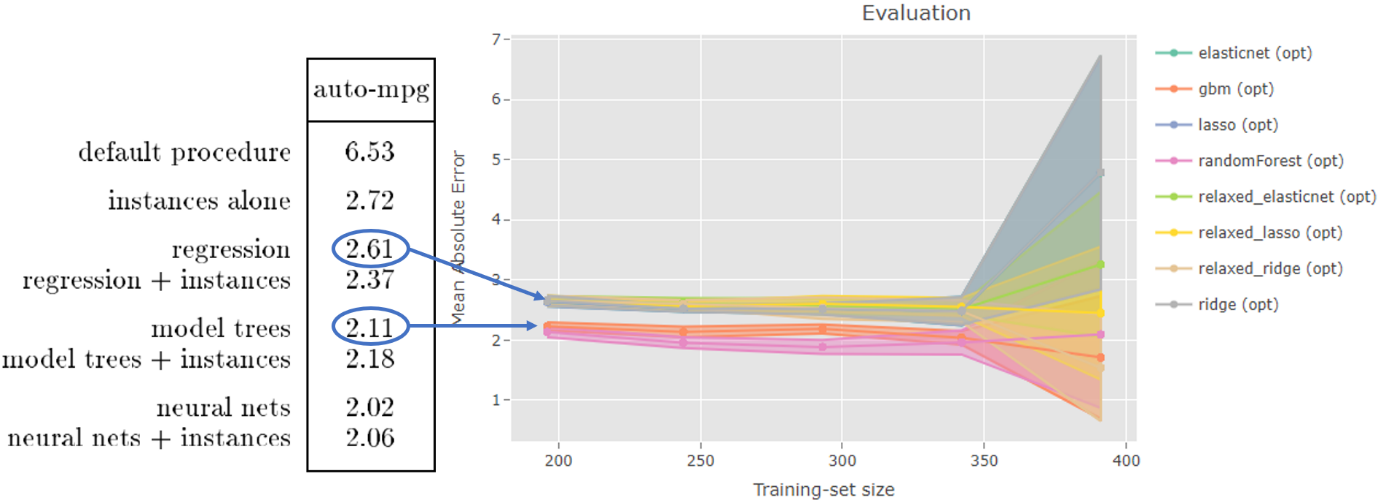


**Use case**

The batch-corrected gene expression data for the GDSC cell lines and the clinical trial were retrieved from https://genome.med.nyu.edu/public/tsirigoslab/deep-drug-response (files bortezomib_cells.rds and bortezomib_clinical.rds), a repository linked with the paper from Sakellaropoulos and colleagues^22^. The IC50 scores used for the training of the models were included in the bortezomib_cells.rds file. Patients’ response to Bortezomib was extracted from the supplementary material of the paper (https://ars.els-cdn.com/content/image/1-s2.0-S2211124719314883-mmc2.xlsx).
